# Why “Where” Matters as Much as “How Much”: Single-Cell and Spatial Transcriptomics in Plants

**DOI:** 10.3390/ijms262411819

**Published:** 2025-12-07

**Authors:** Kinga Moskal, Marta Puchta-Jasińska, Paulina Bolc, Adrian Motor, Rafał Frankowski, Aleksandra Pietrusińska-Radzio, Anna Rucińska, Karolina Tomiczak, Maja Boczkowska

**Affiliations:** 1 Plant Breeding and Acclimatization Institute—National Research Institute, Radzików, 05-870 Błonie, Poland; k.moskal@ihar.edu.pl (K.M.); m.puchta@ihar.edu.pl (M.P.-J.); p.bolc@ihar.edu.pl (P.B.); a.motor@ihar.edu.pl (A.M.); r.frankowski@ihar.edu.pl (R.F.); a.pietrusinska@ihar.edu.pl (A.P.-R.); a.rucinska@ihar.edu.pl (A.R.); k.tomiczak@ihar.edu.pl (K.T.); 2 Botanical Garden, Center for Biological Diversity Conservation in Powsin, Polish Academy of Science, Prawdziwka 2, 02-976 Warszawa, Poland

**Keywords:** single-cell RNA sequencing, single-nucleus RNA sequencing, spatial transcriptomics, single-cell multiomics (RNA + ATAC), protoplasts, nuclear isolation, ambient RNA, doublet detection, plant single-cell atlas

## Abstract

Plant tissues exhibit a layered architecture that makes spatial context decisive for interpreting transcriptional changes. This review explains why the location of gene expression is as important as its magnitude and synthesizes advances uniting single-cell/nucleus RNA-seq with spatial transcriptomics in plants. Surveyed topics include platform selection and material preparation; plant-specific sample processing and quality control; integration with epigenomic assays such as single-nucleus Assay for Transposase-Accessible Chromatin using sequencing (ATAC) and Multiome; and computational workflows for label transfer, deconvolution, spatial embedding, and neighborhood-aware cell–cell communication. Protoplast-based single-cell RNA sequencing (scRNA-seq) enables high-resolution profiling but introduces dissociation artifacts and cell-type biases, whereas ingle-nucleus RNA sequencing (snRNA-seq) improves the representation of recalcitrant lineages and reduces stress signatures while remaining compatible with multiomics profiling. Practical guidance is provided for mitigating ambient RNA, interpreting organellar and intronic metrics, identifying doublets, and harmonizing batches across chemistries and studies. Spatial platforms (Visium HD, Stereo-seq, bead arrays) and targeted imaging (Single-molecule fluorescence in situ hybridization (smFISH), Hairpin-chain-reaction FISH (HCR-FISH), Multiplexed Error-Robust Fluorescence In Situ Hybridization (MERFISH)) are contrasted with plant-specific adaptations and integration pipelines that anchor dissociated profiles in anatomical coordinates. Recent atlases in *Arabidopsis*, soybean, and maize illustrate how cell identities, chromatin accessibility, and spatial niches reveal developmental trajectories and stress responses jointly. A roadmap is outlined for moving from atlases to interventions by deriving gene regulatory networks, prioritizing cis-regulatory targets, and validating perturbations with spatial readouts in crops. Together, these principles support a transition from descriptive maps to mechanism-informed, low-pleiotropy engineering of agronomic traits.

## 1. Introduction

Plant tissues are inherently architectural. Cell identities, interfaces, and long-distance conduits are arranged in layers to control transport and signaling along organ axes, from roots to shoots. In such a system, quantifying the extent of transcript changes is necessary but not sufficient: understanding where those changes occur relative to tissue boundaries, neighbors, and organ axes is often decisive for inferring mechanisms. Over the past five years, single-cell and single-nucleus transcriptomics and spatially resolved transcriptomics (SRTs) have converged to meet this need. Dissociated assays resolve cellular heterogeneity, whereas spatial methods preserve the tissue coordinates required to infer intercellular communication, boundary effects, and developmental axes. Recent plant-specific guidelines have outlined best practices for experimental design, quality control and data integration that explicitly account for the challenges posed by plant tissues.

Single-cell RNA sequencing (scRNA-seq) has transformed plant molecular biology by enabling the analysis of transcriptomes at single-cell resolution allowing the discovery of rare cell types, transient states, and dynamic gene expression patterns within heterogeneous tissues [1,2]. However, tissue dissociation disrupts native spatial organization and leads to a loss of positional information, which is essential for understanding cellular functions and interactions in situ [1,2,3].

Spatial transcriptomics has emerged as a complementary approach that preserves gene expression patterns within intact tissues. Spatial barcodes combined with imaging enable high-resolution transcriptomic maps while maintaining tissue architecture [1,2,3]. This phenomenon is particularly important in plant biology, where positional cues, intercellular signaling, and developmental gradients significantly influence cell identity and function [1,3,4].

Recent studies have begun to apply these methods to model and crop species. Methodological advances now allow single-cell, single-nucleus and spatial assays to be implemented in *Arabidopsis thaliana* and in key crops such as rice, maize, and wheat [4,5,6]. These efforts have generated spatially resolved atlases that reveal cellular heterogeneity and gene regulatory networks across tissues and developmental stages [5,7]. For example, spatial mapping of roots has delineated anatomical domains and functional zones, including meristematic, vascular, and epidermal layers, and revealed expression gradients critical for root growth and nutrient uptake [5,7]. Similarly, spatial transcriptomics of leaves revealed cell type-specific transcriptional responses to drought and osmotic stress [8,9].

Integrating spatial transcriptomics with single-cell data provides new insights into developmental pathways and intercellular communication networks, bridging the gap between the quantitative expression of transcripts (“how much”) and their spatial distribution (“where”) [1,4,10]. Because spatial context shapes regulatory networks and cellular function, such integrative analyses are particularly powerful for understanding plant development and environmental adaptation [3,4]. Moreover, spatially resolved transcriptomics accelerates crop improvement by identifying gene networks that regulate traits such as stress tolerance, growth, and metabolite biosynthesis, thereby highlighting targets for precision breeding and genetic engineering [9,11]. Deciphering these spatial gene regulation circuits allows the identification of new targets for precision breeding and genetic engineering, which is essential for agricultural research.

This review explains why the spatial dimension of gene expression—“where” transcripts are detected within plant tissues—is as important as absolute abundance—“how much”. We synthesize advances in single-cell and single-nucleus transcriptomics with spatial methods and discuss plant-specific challenges (e.g., protoplasting artifacts, ambient RNA, organellar reads and polyploid mapping) together with practical solutions. Finally, we outline a path for translating atlases into interventions: deriving cell-type gene regulatory networks and cis-regulatory maps, prioritizing edit targets, validating them in situ with spatial readouts, and deploying delivery routes that accelerate iteration in crops. Our aim is to provide design principles and a practical toolkit to enable researchers to move from descriptive maps to precise, low-pleiotropy modifications of agronomic traits.

## 2. Single-Cell Transcriptomics in Plants

### 2.1. Platforms and Material Preparation

Single-cell transcriptomics has become a fundamental methodology in plant molecular biology, enabling the investigation of cellular heterogeneity and transcriptional regulation. The main approaches include single-cell RNA sequencing via enzymatically isolated protoplasts and single-nucleus RNA sequencing (snRNA-seq) via isolated nuclei. The technical considerations of these platforms influence the representation of the transcriptome, the coverage of cell types, and the compatibility with epigenomic assays such as Assay for Transposase-Accessible Chromatin using sequencing (ATAC-seq) (Table S1).

#### 2.1.1. Protoplast-Based scRNA-seq: Opportunities and Limitations

Enzymatic removal of the plant cell wall is the most direct and widely used approach for accessing cytoplasmic mRNAs for scRNA-seq. Protoplast isolation protocols have been optimized to yield viable, intact cells that are suitable for droplet-based transcriptomic profiling, routinely capturing 3000–5000 unique transcripts per cell [12,13]. This resolution allows the identification of subtle metabolic and developmental gradients within meristematic tissues, where cellular heterogeneity critically influences organogenesis and growth regulation [14]. However, the cellulase–pectinase digestion required to degrade rigid plant cell walls constitutes a strong abiotic stimulus. Comparative bulk RNA-seq analyses have shown that protoplasting rapidly induces canonical wound- and stress-responsive gene expression, with thousands of genes upregulated within minutes of incubation [13,15]. Such artifactual signatures must be accounted for during clustering and cell-type assignments to avoid confounding transcriptomic analyses [14].

Another critical limitation is the differential release of various cell types due to their varying susceptibility to enzymatic digestion. Lignified and otherwise recalcitrant cells, such as mature xylem vessels, and sclerenchyma cells, are often underrepresented or missing in protoplast preparations, leading to disparities in sample composition and biases in transcriptomic evaluations [12,15]. Undoubtedly, such selective cell loss complicates atlas construction in tissues with highly heterogeneous cellular architectures [14]. To obtain high-quality protoplast yields while reducing stress-induced artifacts, enzyme mixtures and digestion times must be optimized on the basis of tissue- and species-specific differences in cell wall composition [13]. Recent methodological advances address these challenges by refining enzyme formulations, digestion protocols, and downstream RNA stabilization techniques.

#### 2.1.2. SnRNA-seq: A Solution for Recalcitrant Tissues

SnRNA-seq has emerged as a robust alternative to scRNA-seq based on protoplasts, particularly for plant tissues that are difficult to digest enzymatically because of their rigid secondary cell walls and high metabolite content. This approach avoids the need for enzymatic degradation of the cell wall by isolating intact nuclei directly in detergent-based buffers that facilitate their encapsulation and subsequent processing without requiring whole-cell dissociation [16,17]. Compared with those of the matched protoplast-derived scRNA-seq libraries, the recovered populations increased by two- to four-fold. Moreover, compared with protoplast-based techniques [16,18,19], snRNA-seq substantially minimizes dissociation-induced transcriptional artifacts, leading to a greater than 50% reduction in stress response signals [15,16]. Nevertheless, snRNA-seq datasets typically yield 60–80% of the unique molecular identifiers (UMIs) achieved in protoplast scRNA-seq experiments, and the cell-type identities and transcriptional profiles obtained by the two methods display high concordance [15,16,18]. Nuclear preparation more faithfully preserves native tissue composition, avoiding the biases and viability issues that often plague protoplast-based workflows and can selectively exclude specific cell populations [17,20].

An additional advantage of snRNA-seq in plants is that it is compatible with multiomics platforms. Notably, the 10× Genomics Chromium Single-cell Multiome ATAC + Gene Expression assay enables the simultaneous profiling of chromatin accessibility and gene expression within the same nucleus [4,21]. As a result, snRNA-seq has emerged as the preferred methodology for complex plant tissues—such as woody stems, mature embryos, and secondary xylem—for which protoplast isolation is technically challenging or prone to substantial biases [15,16]. 

#### 2.1.3. Compatibility with Chromatin Assays and Emergence of the Multiome

A key advantage of isolating nuclei for single-cell transcriptomics is their natural suitability for concurrent chromatin accessibility profiling, enabling integrated epigenomic and transcriptomic analyses at single-cell resolution. The 10× Genomics Chromium Single-cell Multiome ATAC + Gene Expression platform exemplifies this synergy, by generating perfectly matched RNA and ATAC libraries from individual nuclei tagged with a unified cellular barcode, thereby allowing the direct correlation of gene expression and chromatin accessibility within the same cellular context [21]. For example, multiome profiling of *Arabidopsis* root tips under osmotic stress revealed early activation of dehydration-responsive element (DRE) motifs exclusively in endodermal nuclei preceding the transcriptional activation of ABA-dependent genes in neighboring cortex cells [21]. The spatiotemporal analysis provides a causal view of regulatory information that could previously be inferred only indirectly by integrating separate scRNA-seq and scATAC-seq datasets. When applied to crop species, such as soybean, an integrative single-nucleus multiome atlas identified over 100 distinct cell types on the basis of combined chromatin accessibility and gene expression signatures, elucidated chromatin regulatory landscapes, and revealed seed-specific enhancer clusters that can be used to reliably predict the onset of oil biosynthesis programs [22]. These findings underscore the utility of multiome data for identifying functional cis-regulatory modules critical to agronomic trait regulation.

In practice, the 10× Chromium Multiome workflow starts with gentle nuclear isolation in a nonionic detergent buffer, preserving nuclear morphology and preventing clumping or debris formation [23,24]. Accessible chromatin regions are then tagmented with Tn5 transposase, while polyadenylated RNA transcripts undergo in-nucleus reverse transcription [25,26]. Both reactions occur inside microfluidic gel beads-in-emulsion (GEMs), where each nucleus is uniquely indexed with cell-specific barcodes and UMIs that tag ATAC and cDNA fragments [25,27]. After separate library amplification and parallel sequencing, matched chromatin accessibility datasets and gene expression datasets are obtained and linked through shared barcodes, enabling integrative single-cell multiomics analyses that reveal regulatory relationships, particularly under dynamic environmental conditions [21,28]. Joint profiling of chromatin and transcriptomic data in the same nucleus supports the reconstruction of transcriptional regulatory networks, the association of peaks with genes, and the identification of cell type-specific regulatory elements with high accuracy [21,27]. As emphasized in vendor protocols and experimental best practices, data quality critically depends on optimized nucleus isolation, high nuclear purity and minimal contamination with intact cells or debris [18].

#### 2.1.4. Best-Practice Recommendations

**Figure 1:**
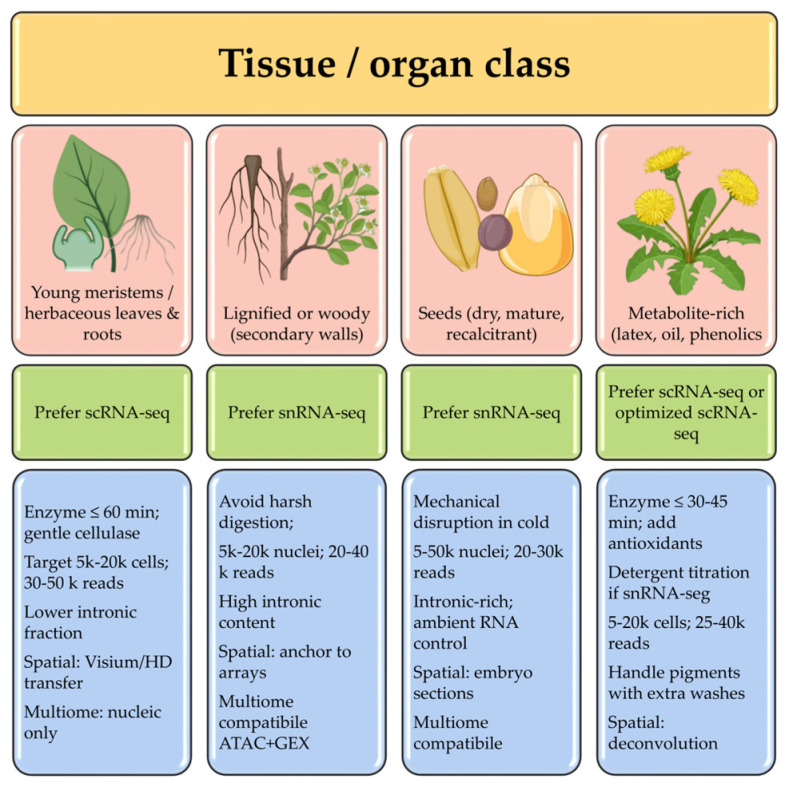
Decision guide for choosing scRNA-seq versus snRNA-seq across major plant tissue classes. Schematic overview of recommended single-cell modalities and key experimental considerations for different plant organs. From left to right, cartoon icons depict: (i) young meristems and herbaceous leaves/roots with thin primary walls; (ii) lignified or woody tissues with extensive secondary wall deposition; (iii) dry or mature seeds and other highly recalcitrant organs; and (iv) metabolite-rich tissues containing latex, oils, or phenolics. For each tissue class, the middle row summarises the preferred approach (protoplast-based scRNA-seq, snRNA-seq, or optimized scRNA-seq), whereas the bottom row lists practical guidelines, including recommended digestion or disruption conditions, approximate target cell/nucleus and read depths, expected intronic content and ambient RNA burden, and compatibility with spatial or multiome assays. Together, the schematic provides a rapid, practice-oriented roadmap for selecting and tuning single-cell workflows in diverse plant materials. This figure was edited with BioRender (accessed via web application on app.biorender.com).

Recent consensus guidelines provide a comprehensive decision tree for plant single-cell transcriptomics, summarizing tissue-specific factors, technical limitations, and key data quality criteria [18]. Protoplast-based scRNA-seq is recommended for embryonic tissues, callus cultures, and young meristems in which enzymatic digestion can be completed rapidly, minimizing dissociation-induced stress-response artifacts. For more differentiated tissues with substantial secondary cell wall deposition, such as mature leaves and seeds, snRNA-seq is recommended because protoplast isolation is technically challenging or biased against certain cell types (Figure 1). Nuclear preparation avoids enzymatic wall digestion and enables transcriptomic profiling of recalcitrant tissues that are difficult to dissociate into protoplasts [1,18]. A hybrid approach, in which protoplasts are isolated from easily digestible peripheral layers and more recalcitrant inner tissues, offers a practical compromise for complex organs. Advanced computational frameworks such as Harmony and Scanorama are then used to correct for platform- and batch-specific effects and embed the combined datasets in a shared latent space, improving comparability across experiments and genotypes [29,30]. Best-practice guidelines also emphasize several quality control (QC) checkpoints. For droplet-based assays, ambient RNA contamination should be estimated and corrected with tools such as SoupX or FastCAR [31,32]; chloroplast-derived UMI fractions require tissue-specific thresholds to exclude dying or damaged cells; and doublet prediction (for example, via Scrublet or Solo) is essential to prevent artifactual merging of transcriptomes [33,34]. In snRNA-seq and multiome libraries, intronic reads should be retained and handled explicitly, reflecting the prevalence of nascent nuclear transcripts [16]. For multiome assays, biological replicates are essential because chromatin accessibility profiles show greater intrinsic variability than transcript abundance does. Rigorous batch correction and integrated analysis are needed for robust peak-to-gene linkage and reconstruction of transcriptional regulatory networks [2,21].

#### 2.1.5. Emerging Technologies and Future Directions

Progress in plant single-cell transcriptomics is driven by innovations that reduce artifacts and enable multimodal measurements. Compared with protoplasting, enzyme-free mechanical dissociation with nanobody-based nuclear capture avoids enzymatic wall digestion, preserves nuclear integrity and limits stress–response artifacts [1]. Droplet microfluidic systems provide high- throughput nuclear encapsulation, increasing the scalability and sensitivity of snRNA-seq [18]. Pilot studies combining snRNA-seq with single-nucleus Hi-C indicate that concurrent profiling of transcription, chromatin accessibility, and three-dimensional genome topology in individual nuclei will be feasible [21,35]. Until trimodal platforms became routine, joint snRNA-seq and ATAC + gene expression multiome assays remained the state-of-the-art methods for reconstructing gene regulatory networks and linking the chromatin state to transcription, including transcription factor binding, enhancer–promoter contacts and cell type-specific epigenetic regulation [4,21]. Using integrated methods to analyze plant cell heterogeneity and adaptive response mechanisms facilitates basic research and advances translational breeding. These integrated readouts, together with evolving bioinformatics toolkits for multimodal integration and spatial mapping, are reshaping plant biology by revealing cellular identity and developmental plasticity and by providing targets for translational breeding [3,4].

### 2.2. Quality Control and Plant-Specific Artifacts

**Figure 2:**
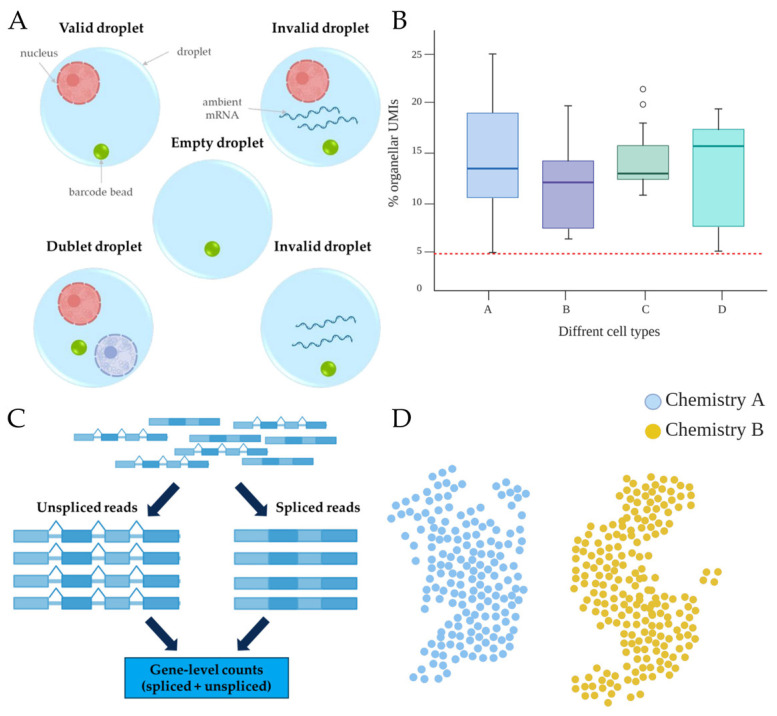
Quality control and plant-specific artifacts in single-cell and single-nucleus transcriptomic data. (**A**) Schematic outcomes of droplet-based capture, including a valid droplet containing a single nucleus and barcode bead, an empty droplet containing only ambient mRNA, a doublet droplet with two nuclei, and droplets with degraded material or ambient RNA only. These scenarios illustrate the need to identify and correct for ambient background and doublets during preprocessing. (**B**) Example distributions of organellar UMI fractions across four plant cell types (**A**–**D**). The red dashed line marks the commonly used 5% mitochondrial read threshold from animal QC workflows; in plants, plastid-rich cell types (e.g., bundle sheath-like populations) can greatly exceed this value under normal physiology, emphasizing the need for cell-type-aware, plant-specific thresholds. (**C**) Conceptual view of intronic (unspliced) and exonic (spliced) reads mapped to a gene model. Separate quantification of unspliced and spliced reads before aggregation into gene-level counts preserves nuclear pre-mRNA and supports advanced analyses such as RNA velocity and pseudotime inference. (**D**) Illustration of batch effects, in which the same underlying cell populations profiled with two different chemistries (Chemistry A and Chemistry B) form separated manifolds prior to integration, highlighting the requirement for dedicated harmonization methods to align batches while maintaining biological structure. This figure was edited with BioRender (accessed via web application on app.biorender.com).

The quality of molecules entering the sequencing pipeline is critical for the informativeness of high-resolution single-cell datasets. In plants, QC is complicated by rigid cell walls, abundant plastids and vacuoles, and the need to integrate data from protoplast-, single-nucleus-, multiome-, and spatial transcriptomics. These features require plant-tailored QC protocols that address four key domains: ambient RNA contamination, organellar transcript content, doublet detection, and batch-effect correction (Figure 2).

#### 2.2.1. Ambient RNA

Ambient RNA contamination, arising from lysed protoplasts or ruptured nuclei whose transcripts are co-captured by neighboring droplets, is a pervasive challenge in droplet-based single-cell and single-nucleus RNA-seq [31,36]. Even low contamination rates (1–2%) can affect housekeeping gene expression, homogenize cellular profiles and obscure true heterogeneity, thereby confounding clustering analyses [31,36]. In plants, this problem is exacerbated by enzymatic cell wall digestion, which promotes cytoplasmic mixing, and by abundant plastid-derived ribosomal RNAs that are often polyadenylated and consequently captured during library preparation [2,18]. To mitigate ambient RNA contamination, computational correction is routinely applied. SoupX estimates an ambient “soup” profile from low-UMI barcodes and subtracts this background gene by gene from each cell, effectively reducing systematic contamination [31]. FastCAR uses an expectation–maximization framework to infer both cell-type assignments and per-cell contamination fractions jointly, improving cluster separation in complex tissues [32]. Despite these advances, three user decisions critically determine correction quality, i.e., defining an appropriate background gene set, which differs between nucleus- and protoplast-derived libraries because their ambient RNA sources are distinct; preserving truly low-abundance transcripts to avoid overcorrection and loss of biological signals; and recalibrating cluster marker statistics after decontamination to prevent overfitting and spurious confidence in cell-type markers [1,18]. Careful handling of these steps is essential for rigorous QC and reliable interpretation of plant single-cell and single-nucleus transcriptomic data.

#### 2.2.2. Organellar and Intronic Metrics

In scRNA-seq analyses, data quality is often assessed by the fraction of reads mapped to organellar genomes and the proportion of intronic reads. Thresholds derived from animal datasets, where cells with >5% mitochondrial reads are typically flagged as low quality or apoptotic, are not directly applicable to plants because of abundant plastids [36]. For example, single-cell data from *Nicotiana tabacum* leaves revealed a median UMI chloroplast fraction of ~14% and values > 40% in bundle sheath cells, with evidence of cellular stress or apoptosis [14]. In plant tissues, plastid read fractions should therefore be evaluated in a cell-type-aware manner, for example, by flagging outliers only when plastid-derived transcripts exceed three standard deviations above the cluster-specific median, reflecting genuine biological variability between cell types rather than damage [18]. Intronic reads predominantly correspond to nuclear pre-mRNAs and unspliced transcripts [16]. Filtering solely on exon UMIs disproportionately removes valid nuclear transcripts and can distort downstream analyses [20]. A widely adopted strategy is to quantify spliced and unspliced reads separately and then combine them to obtain gene-level counts. This preserves biological signals while enabling advanced analyses such as RNA velocity and pseudotime inference, which explicitly exploit information contained in unspliced fractions [37,38]. Therefore, incorporating plant-specific organellar and intronic metrics into QC pipelines is essential for accurate interpretation of plant single-cell and single-nucleus datasets and for robust peak-to-gene linkage and reconstruction of transcriptional regulatory networks.

#### 2.2.3. Doublet Detection

Doublets arise when two or more cells or nuclei are co-encapsulated in a single droplet, producing artifactual transcriptomes that inflate apparent cellular diversity and distort trajectories and lineage inference [33,39]. Simulation-based tools such as Scrublet and DoubletFinder can achieve false-negative rates below 5% when cell types are well balanced [33,39]. However, their sensitivity decreases for doublets involving rare or transcriptionally similar populations which is an important limitation in plant tissues with developmental continua and scarce cell types [40,41]. Recent methods based on deep neural networks, including Vaeda and scMODD, jointly model transcriptomic features and doublet likelihood and recover true doublets with higher sensitivity and specificity, particularly in heterogeneous plant nuclei datasets [1,42,43]. The best practice is a two-tiered strategy: initial removal of obvious multiplets with Scrublet at a liberal threshold reflecting the expected doublet rate (~0.8% per 1000 protoplasts or ~1.2% per 1000 nuclei), followed by refined reclassification of the remaining cells via a deep learning-based classifier to maximize sensitivity without compromising precision [33,42]. When feasible, combining these computational approaches with experimental strategies such as cell hashing or sample multiplexing further reduces the doublet burden and improves the interpretability and reliability of plant single-cell and single-nucleus transcriptomic data.

#### 2.2.4. Batch Effects and Harmonization

Batch effects are major technical confounders in scRNA-seq and related multiomics assays, arising from differences in enzymes, isolation buffers, instrumentation, personnel and tissue physiology across experiments. These nonbiological variations can mask true signals or create artificial patterns that bias clustering, differential expression, and trajectory analyses [30,44]. Effective batch correction is therefore essential when combining data across experiments, laboratories, or chemistries. Linear methods operating in principal component analysis (PCA) space, such as Harmony, scale to datasets with over one million cells and have been used to successfully merge complex series, including ten developmental stages of the *Arabidopsis thaliana* life-cycle atlas, while preserving biological variance [45]. More recently, probabilistic latent variable models, such as single-cell variational inference (scVI), have outperformed linear approaches when heterogeneous chemistries are integrated, for example, when protoplast- and single-nucleus-based datasets are combined in plant consortia [46]. A 2024 benchmark across plant and animal datasets identified scVI as providing superior batch mixing (lowest kBET rejection) and the best preservation biological identity (highest silhouette scores) [46]. Scvi-hub now distributes pretrained models for major plant atlases, lowering the barrier to robust batch correction and enabling reproducible, multi-experiment meta-analyses in plant single-cell transcriptomics [46,47].

### 2.3. Resources and Recent Syntheses

#### Databases and Interactive Portals

The rapid expansion of plant single-cell transcriptomic data has prompted the development of dedicated databases and interactive portals to support data exploration, comparative analysis, and functional annotation across species. A pioneering resource is scPlantDB, the first cross-species plant single-cell database, which currently hosts ~2.4 million sc/snRNA-seq profiles from 17 species, and provides an expression browser, trajectory viewer, and orthologous marker group (OMG) module to trace homologous cell types across lineages [48]. Complementary marker-focused resources include PlantscRNAdb v4, which integrates single-cell and bulk evidence to curate ~59,000 experimentally supported marker genes from 33 plant species, and the Plant Cell Marker Database (PCMDB), which catalogs over 81,000 genes annotated to 263 cell types across six species, thereby linking genomic signatures to cellular contexts and developmental states [49,50,51]. Newer resources such as GreenCells specialize in long noncoding RNAs (lncRNAs) integrating atlases from eight angiosperm species and assigning >2000 lncRNAs to conserved cell states, thus illuminating regulatory layers beyond protein-coding genes [51]. The leading plant single-cell atlases are increasingly accompanied by interactive portals. The *Arabidopsis thaliana* life-cycle atlas provides > 400,000 nuclei across ten stages with stage-resolved uniform manifold approximation and projection (UMAP) embeddings and configurable expression heatmaps for the dynamic exploration of developmental trajectories and similar tools now accompany maize and soybean atlases [45]. Collectively, these databases and portals democratize access to complex plant single-cell datasets, enabling reproducible, comparative analyses across taxa and developmental contexts and providing critical infrastructure for hypothesis generation in plant biology.

### 2.4. Key Takeaways

- Plant single-cell transcriptomics relies on two platforms: protoplast-based scRNA-seq (high cytoplasmic resolution, but prone to stress artifacts and cell-type bias) and nucleus-based snRNA-seq (less prone to artifacts, better for lignified and complex tissues, but with slightly lower UMI depth).
- snRNA-seq is compatible with a single-nucleus multiome (ATAC + gene expression), enabling direct linkage of chromatin accessibility to transcription and facilitating the discovery of cis-regulatory modules relevant to agronomic traits.
- Emerging technologies, such as enzyme-free mechanical dissociation, high-throughput droplet systems, and pilot triomics assays (RNA + ATAC + Hi-C), are enabling the exploration of new questions in more diverse tissues and species.
- Rigorous quality control for plants is essential. It must address ambient RNA, organellar and intronic read fractions, doublets, and batch effects, using appropriate computational tools and plant-specific thresholds.
- Dedicated databases and portals (e.g., scPlantDB, PlantscRNAdb, PCMDB, GreenCells, and species-specific atlases) provide curated markers and user-friendly interfaces.

## 3. Spatial Transcriptomics in Plants—Technologies and Adaptations

Spatial transcriptomics transforms plant biology by enabling gene expression analysis within the native tissue context and preserving tissue architecture. Unlike scRNA-seq, spatial transcriptomics preserves positional information and allows researchers to study the influence of spatial localization on cellular identity and function [4,52].

### 3.1. Capture-Based Sequencing Platforms for Spatial Transcriptomics in Plants

Sequencing-based capture arrays are the workhorses of plant spatial transcriptomics, combining transcriptome-wide coverage with simple library chemistry. The three principal platforms are contrasted below. The plant-specific adaptations and analytical pipelines used are outlined in Table S2.

#### 3.1.1. 10× Visium HD: Near-Cellular Resolution on Standard Slides

The original 10× Genomics Visium assay uses 55 µm barcoded spots, which are often too coarse for finely patterned plant tissues. Visium HD, introduced in 2024, replaces these spots with a continuous lawn of densely packed 2 µm oligonucleotide tiles, increasing feature density by ~two orders of magnitude and enabling near single-cell resolution [53]. Each tile acts as an independent capture probe and is barcoded according to a Hilbert curve layout, allowing automated reassembly of cellular masks and enabling near-single-cell resolution. In the FFPE mouse brain, this yields a median of ~8000 UMIs per 100 µm^2^, representing a substantial resolution gain over conventional Visium while maintaining compatibility with standard histology [53]. These advances enable precise spatial gene expression mapping while maintaining compatibility with standard histological preparation. Adaptations of plant tissues consider their unique physiology. In *Arabidopsis thaliana* rosette leaves and shoot apices, FFPE sections require extended permeabilization (~16–18 min) to ensure efficient RNA release [54]. Pilot datasets from meristematic tissues reveal the classical epidermal, L2, and L3 layers and localize auxin-responsive transcripts to incipient primordia, illustrating the capacity of Visium HD to capture biologically meaningful spatial patterns. At this scale, downstream analysis can adopt cell-level workflows, including label transfer from sc/snRNA-seq (e.g., Seurat v5), image-based cell segmentation (e.g., CytAssist pipelines) and ligand–receptor interaction inference constrained by physical adjacency [54]. Visium HD also imposes practical constraints. A single 6 × 6 mm slide can be generated on the order of 10^7^ barcodes and ~250 GB of raw data, necessitating robust computational and storage infrastructure. In photosynthetic tissues, abundant chloroplast RNA can dominate the signal; incorporating RNase H during sample clarification has been shown to mitigate this issue and improve the specificity of nuclear transcript capture [54].

#### 3.1.2. Stereo-seq: Subcellular Resolution and Centimeter-Scale Fields

Stereo-seq (Spatial Enhanced Resolution Omics-sequencing) combines spatial resolution with centimeter-scale fields of view via DNA nanoball-patterned chips with a 500 nm pitch [55]. In practice, adjacent nanoballs are grouped into 0.5–1 µm “spotlets” that form the basic spatial units for transcript capture, enabling detailed mapping of transcriptomes within individual plant cells over large tissue areas. A recent application to maize ear development generated a 6 × 6 mm spatial map with >1.2 billion reads and resolved 12 spatially ordered cell types, including four previously uncharacterized populations [56]. This capture area exceeds that of traditional Visium, making Stereo-seq especially suitable for whole-organ analyses of structures such as caryopses, siliques, and tubers. Plant-specific protocols typically employ 8–10 µm cryosection and methanol fixation to preserve RNA and ensure reagent diffusion across nanoball arrays, whereas hydrophobic chip surfaces require custom silicone gaskets to prevent tissue curling during mounting. Vendor pipelines (e.g., STOmics) support binning, quality control, pseudo-cell aggregation, and label transfer from scRNA-seq using algorithms such as Tangram or CellTrek. At near −1 µm resolution, Stereo-seq can resolve fine spatial gradients in gene expression across storage tissue and meristems, providing unprecedented detail about cellular organization and metabolic patterning within plant organs.

#### 3.1.3. Bead-Array Alternatives: Slide-seq v2 and HDST

Slide-seq v2 uses a randomly packed array of 10 µm DNA-barcoded beads attached to rubber-coated glass to achieve near single-cell spatial resolution [57,58]. Random bead placement provides flexible resolution but requires computational decoding to reconstruct spatial maps. This approach is a cost-effective alternative to commercial platforms such as 10× Genomics Visium and can be chemically customized, for example, to capture full-length transcripts or specific RNA modifications. High-definition spatial transcriptomics (HDSTs) extends this concept by printing 2 µm DNA-barcoded beads into lithographically defined, addressable wells, yielding markedly higher spatial resolution than Slide-seq v2 [59]. The ordered layout reduces spatial uncertainty and improves transcript localization, which is advantageous for finely patterned plant tissues. Because bead arrays are typically performed under sample transcripts, resolution-enhancement algorithms such as BayesSpace use a Markov random field to borrow information from neighboring beads and estimate sub-spot expression, improving boundary detection and spatial domain delineation by ~25% in Slide-seq data from *Arabidopsis* ovules [60]. As a Bioconductor package, BayesSpace supports data from Visium, Slide-seq, HDST, and Stereo-seq bins, facilitating refined spatial analyses [60]. The remaining challenges include bead misregistration and imperfect bead–tissue contact. Mosaic-seq strategies that photolithographically barcode both beads and tissue sections can reduce decoding errors to <0.5%, whereas clearing agents such as ScaleA2 and adhesion-promoting peptides improve bead–tissue apposition in thick plant sections, limiting RNA diffusion and increasing capture fidelity.

### 3.2. Targeted Imaging-Based Methods for Plant Spatial Transcriptomics

Although sequencing-based capture platforms yield atlas-scale coverage, their resolution rarely exceeds the cellular level, and they require substantial RNA input. Targeted imaging approaches, such as fluorescence in situ hybridization (FISH) and in situ sequencing (ISS), complement capture arrays by providing single-molecule sensitivity, subcellular resolution, and flexible gene panels that can validate or extend findings from dissociated single cells.

#### 3.2.1. smFISH and HCR-FISH in Whole-Mount Tissues

Single-molecule fluorescence in situ hybridization (smFISH) achieves transcript-level resolution by tiling ~48 fluorophore-coupled oligonucleotides along a target mRNA, producing diffraction-limited puncta that correspond to individual molecules [61]. Adapting smFISH to intact plant organs requires enzymatic softening of the pectin-rich cell wall to allow the probes to enter; second, optical clearing via ClearSee to quench chlorophyll autofluorescence; and refractive index matching to minimize light scattering in curved tissues [62]. Zhao et al. used mild pectolyase pretreatment and ClearSee to visualize whole-mount smFISH patterns in *A. thaliana* roots, localizing *WOX5* puncta to the quiescent center and SCR puncta to endodermal initials without sectioning while preserving fluorescent protein reporters for simultaneous RNA–protein quantification [63].

Hairpin-chain-reaction FISH (HCR-FISH) replaces enzymatic amplification with a self-assembling polymer of DNA hairpins triggered by a target-bound initiator, generating bright, diffraction-limited spots, without RNase treatment associated with tyramide systems [64]. In maize leaf primordia, Huang et al. optimized whole-mount HCR to multiplex twelve developmental regulators in a single sample with ~2 µm axial resolution, revealing fine proximodistal expression gradients that are apparent in the bulk or dissociated data [65]. Because HCR does not require complete wall digestion, it is well suited to thick or partially lignified organs, such as cereal grains, siliques, and fleshy fruits.

Together, smFISH and HCR-FISH form a complementary toolkit. smFISH provides an exact measurement of low-copy transcripts identified by scRNA-seq, whereas HCR-FISH scales to 10–20 gene panels for rapid screening of regulatory circuits across developmental axes or treatment gradients [4]. These imaging modalities provide the single-molecule “ground truth” for capture-based spatial atlases, which anchor dissociated-cell prediction in their correct microscopic context and bridge high-throughput sequencing with cell-resolved developmental biology.

#### 3.2.2. MERFISH: Combinatorial Barcoding at Scale

Multiplexed Error-Robust Fluorescence In Situ Hybridization (MERFISH) assigns error-tolerant binary barcodes to RNA targets, which are decoded via sequential hybridization and imaging, enabling the quantitative detection of hundreds to thousands of transcripts at single-molecule resolution in intact tissues [66,67]. MERFISH has been implemented in plants since 2025. The *Arabidopsis thaliana* life-cycle atlas integrates snRNA-seq and spatial assays to analyze MERFISH datasets across organs whereas resistance studies combined the snMultiome and MERFISH datasets to localize rare resistant cells in infected leaves, illustrating its value for mapping specialized niches in vivo [45,68]. Plant MERFISH still requires tissue-specific optimization, i.e., partial wall digestion, permeabilization and suppression of autofluorescence, and no single pretreatment protocol yet generalizes across organs, thus empirical tuning remains necessary. Panel design is best guided by markers from sc/snRNA-seq (e.g., cell type-specific transcription factors or transporters), allowing MERFISH to validate dissociation-based annotations and refine spatial boundaries. For very low-abundance targets, orthogonal imaging methods such as whole-mount HCR-FISH, which was recently optimized for plants, provide complementary sensitivity while remaining chemically distinct from MERFISH [65,67].

#### 3.2.3. ISS and Padlock-Probe Chemistries

Padlock probe-based spatial transcriptomics (ISS) is an imaging-based spatial transcriptomics modality in which target mRNAs are reverse-transcribed and resulting cDNA serves as template for padlock probes. After circularization and rolling-circle amplification (RCA), submicron “rolonies” are generated and decoded via iterative fluorescent readouts. Variants such as HybISS and workflows such as BaristaSeq have been extensively validated in mammalian tissues. Although large-panel in situ hybridization (ISH) applications in plants remain limited, the same principles apply, provided that cell wall permeability and background autofluorescence are carefully controlled, and mild protease and permeabilization steps are combined with mechanical stabilization (e.g., agarose co-embedding) to enhance probe penetration while preserving RNA and section integrity [69,70]. Compared with MERFISH, ISS generally provides larger gene panels per run but offers lower per-gene sensitivity and involves more complex library construction Panel design is therefore critical. Selecting markers from dissociation-based atlases (e.g., Visium-anchored differential or co-expression modules identified by WGCNA/hdWGCNA) increases the biological informativeness of targets and facilitates cross-validation of cell-type annotations and tissue domains. Recent computational frameworks (e.g., GPS-FISH) formalize this design step and improve sensitivity to relevant cell states [71,72,73]. As plant-optimized protocols and community resources mature, padlock-based ISS is poised to complement capture-based and FISH-based spatial approaches in plant systems [71].

#### 3.2.4. Validation and Integration Pipelines

Targeted imaging plays two indispensable roles in plant spatial genomics, i.e., orthogonal validation of hypotheses from capture-based (e.g., Visium/Stereo-seq) and dissociation-based (e.g., sc/snRNA-seq) assays, and fine-scale extension where sequencing reaches its spatial limits. Whole-mount smFISH and multiplex HCR-FISH, which were established in *Arabidopsis* and cereals, provide single-molecule measurements and multigene panels in intact organs, enabling validation of cell-type markers and refinement of domain boundaries [65,74]. In maize, a Stereo-seq atlas of the developing ear used imaging-level assays to confirm spatially organized meristem states and candidate markers, illustrating how imaging corroborate dcapture-based maps [56]. A typical integration workflow comprises three steps: spot calling, segmentation and molecule assignment, and co-registration to capture arrays. FISH-Quant v2 and starfish support point-spread-function-aware detection, the deconvolution of dense clusters, decoding multi-round imaging and the export of per-molecule coordinate tables for downstream analyses [74,75]. Plant tissues benefit from membrane/wall stains and nuclei counterstains; deep-learning tools such as CellPose and CellPose 2.0 and plant-specific pipelines such as PlantSeg generate cell masks from confocal stacks, whereas Baysor performs probabilistic cell delineation directly from molecular coordinates and has become the standard for assigning transcripts to cells in imaging-based datasets [76,77,78]. Deformable registration with ANTs, which uses mutual information or landmark-driven transforms, is widely applied to align fluorescence images with Visium or Stereo-seq coordinates and to achieve pixel/spot-level correspondence across modalities [79]. These pipelines enable quantitative comparisons between imaging-derived counts and capture-based expression matrices, increase confidence in cell type annotations, sharpen boundaries beyond array well size, and refine ligand–receptor neighborhoods. As plant atlases proliferate, such integrated imaging-spatial workflows are becoming indispensable for building and testing models of development and stress responses [56].

### 3.3. Plant-Specific Adaptations

Cryosections of 10 ± 2 µm are generally used in plant spatial studies, with the thickness adjusted to each organ to balance diffusion and section integrity. Studies focusing on embryos have reported optimized permeabilization times, such as 6 min at 37 °C, to maintain spatial sharpness [80,81]. Cross-platform benchmarking supports conservative chemistry, as excessive molecular diffusion during permeabilization can compromise cellular resolution [82]. Although pre-permeabilization with cell wall enzymes is common in imaging assays, no peer-reviewed evidence supports it as a standard step for plant Visium work. Instead, plant protocols emphasize organ-specific optimization, compatible staining, and careful handling to protect RNA [81,83]. Tailored workflows continue to emerge; for example, the 2025 STAR Protocols describe standardized methods for soybean Visium analyses, from sample preparation to permeabilization [84]. Key adaptations from animal to plant studies include tuning permeabilization, adjusting section thickness/orientation, and incorporating diffusion-aware analysis.

### 3.4. Key Takeaways

- Capture-based platforms (10× Visium HD, Stereo-seq, Slide-seq v2/HDST) offer complementary trade-offs between spatial resolution, field of view, cost and data volume; all require plant-specific optimization of section thickness, permeabilization and data processing.
- Visium HD delivers near-cellular resolution on standard slides and is readily integrated with sc/snRNA-seq label transfer, but in plants it demands longer permeabilization, mitigation of chloroplast RNA and substantial computational/storage resources.
- Stereo-seq achieves subcellular resolution over centimeter-scale areas, enabling whole-organ maps (e.g., caryopses, siliques, tubers) and fine expression gradients, at the cost of demanding cryo-sectioning, fixation and specialized vendor pipelines.
- Bead-array methods (Slide-seq v2, HDST) provide flexible and often more affordable alternatives, with HDST improving spatial precision via ordered 2 µm wells; resolution-enhancement tools such as BayesSpace and experimental refinements (mosaic-seq, clearing and adhesion strategies) are critical for high-quality plant data.
- Targeted imaging (smFISH, HCR-FISH, MERFISH, ISS) supplies single-molecule and subcellular readouts that validate markers from sc/snRNA-seq, refine tissue domains and resolve rare or specialized cell states that are difficult to capture with sequencing alone.
- Dedicated integration pipelines (FISH-Quant, starfish, CellPose/PlantSeg, Baysor, ANTs) enable rigorous alignment of imaging and capture data, improving cell-type annotation, boundary sharpness and ligand–receptor neighborhood inference in plant atlases.
- Across technologies, successful spatial transcriptomics in plants hinges on organ- and species-specific adaptations—particularly for wall/cuticle permeability, diffusion control and staining regimes—with emerging standardized protocols (e.g., soybean Visium) beginning to codify best practices.

## 4. Integrating sc/snRNA-seq with Spatial Transcriptomics

Integration aims to recover cell identities and states across tissue space while respecting anatomical constraints and neighborhoods. Contemporary pipelines combine label transfer, spot deconvolution, spatially aware representation learning, cell reconstruction from high-definition data, and neighbor-explicit inference of cell-to-cell communication (CCC).

### 4.1. Mapping Cell Identities

Seurat’s anchor-based integration provides a standard workflow for mapping query profiles onto a reference atlas and transferring discrete labels or continuous “imputation” scores. In practice, a high-quality plant sc/snRNA-seq reference (often nuclear-based to minimize protoplasting artifacts) is used to annotate Visium, Visium HD, or imaging datasets via label transfer, with uncertainty or entropy maps highlighting ambiguous regions at organ boundaries [85]. In the Python ecosystem, Scanpy’s ingestion/projection utilities play a similar role by embedding queries in a reference latent space and assigning labels via k-nearest neighbor (k-NN) classifiers [86]. For imaging-based targets or when continuous trajectories are central, Tangram uses deep learning to align sc/snRNA-seq with spatial modalities (MERFISH, STARmap, smFISH, and Visium), providing gene-by-gene reconstructions and supporting multimodal references [87]. TACCO extends optimal transport to unify transfer and decomposition in a single framework, which is particularly useful when the reference and target differ in terms of technology or data support [88].

### 4.2. Deconvolution: Transforming Mixtures into Cell-Type Abundances and States

Because many capture assays aggregate multiple cells, deconvolution is essential. Robust cell type decomposition (RCTD) models spatial counts as a mixture of reference cell-type profiles and corrects for technology differences, providing reliable proportion estimates [89]. Cell2 location uses a Bayesian framework that “borrows strength” across spots to estimate absolute cell-type abundance and performs well in complex tissues [90]. DestVI (scvi-tools) extends this to continuous variation within each cell class, enabling the detection of type-specific spatial state differences [91]. Stereoscopy offers a negative binomial (NB) model that jointly fits single-cell and spatial data with good speed and interpretability [92]. Benchmarks indicate that methods that explicitly model uncertainty and spatial context (e.g., CARD, cell2location, and Tangram-based tactics) outperform simple regression-based models in accuracy and robustness [93]. Emerging graph- and deep-learning methods, including single-cell resolution deconvolution, further refine local composition and state estimates—an advantage in plant tissues with gradual developmental continua [94,95].

### 4.3. Spatial Toolkits for Pattern Discovery and Image Integration

Squidpy and Giotto are currently the leading analytical toolkits. Squidpy (Python) integrates omics and image analysis, generates spatial plots from coordinate data, quantifies neighborhood enrichment and spatial autocorrelation, and extracts histological features which are useful, for example, for linking expression to cell wall architecture or vascular organization [96]. Giotto (R) provides comparable functionality with extensive visualization and well-documented protocols tailored to common workflows in biology [97,98]. SpatialPCA offers dimensionality reduction that preserves the spatial covariance, supporting trajectory interference and region segmentation in leaves or meristems. Spatial transcriptomics analysis with topic modeling to uncover spatial patterns (STAMPs) applies topic modeling to derive interpretable spatial “topics” and gene modules that often correspond to morphogen gradients or tissue layers [99,100]. The choice of platform typically reflects the programming environment (e.g., Python vs. R), and the project needs, i.e., Squidpy integrates tightly with Scanpy/AnnData, whereas Giotto leverages the R ecosystem of bioinformatics and statistical packages.

### 4.4. Cell Segmentation and Reconstruction from High-Definition Data

At subcellular resolutions (e.g., 2 µm barcodes in Visium HD), default square binning (8 × 8 µm or 16 × 16 µm) can blur domain boundaries in densely packed plant tissues. Bin2Cell reconstructs single-cell objects by combining morphology-based image segmentation with expression similarity, correcting the variability introduced by bin geometry and yielding per-cell count matrices with preserved spatial context and sharper morphology [101]. Building on this concept, ENACT provides an end–to–end pipeline that combines advanced segmentation with Visium HD data to infer cell types across entire tissue sections, which has been validated on synthetic and real datasets [102]. In plant laboratories, segmentation is often initialized from nuclear masks (DAPI/H4) to mitigate chloroplast autofluorescence and then refined with wall stains before assigning per-cell expression. This strategy integrates cleanly with downstream cell-type calling and CCC.

### 4.5. Neighbor-Aware Inference of Cell–Cell Communication

Plant cells form stereotyped neighborhoods (e.g., the quiescent center and the endodermis–pericycle), making spatial CCC methods particularly relevant. COMMunication analysis by Optimal Transport (COMMOT) formulates the CCC as an optimal transport problem, considering ligand/receptor competition and spatial distances. Furthermore, it has been shown to identify directionality- and signaling-regulated genes [103]. SpatialDM applies bivariate Moran’s statistics to detect spatially co-expressed ligand–receptor (L–R) pairs and local “hotspots” and scales to millions of spots [104]. DeepTalk integrates scRNA-seq and spatial data in a deep model to recover CCCs at single-cell resolution with improved specificity, complementing classical L–R scoring [105]. To move from co-expression to functional impact, niche-aware tests such as niche-DE quantify genes whose expression in a given cell type depends on the composition of its local neighborhood, providing a bridge between CCC and downstream transcriptional responses [106].

### 4.6. Key Takeaways

- Integrating sc/snRNA-seq with spatial transcriptomics links high-resolution cell states to their anatomical context, enabling cell-type maps and trajectories that respect tissue architecture and neighborhood structure.
- Label transfer tools (Seurat, Scanpy, Tangram, TACCO) project sc/snRNA-seq references onto spatial data, providing cell-type annotations and continuous state scores while flagging ambiguous regions at tissue boundaries.
- Deconvolution methods (RCTD, cell2location, DestVI, Stereoscope, CARD) transform mixed spatial spots into cell-type abundances and states; models that explicitly incorporate uncertainty and spatial context outperform simple regression-based approaches.
- Spatial toolkits (Squidpy, Giotto, SpatialPCA, and STAMP) support pattern discovery, dimensionality reduction and image integration, revealing spatial gene modules that often coincide with morphogen gradients and tissue layers.
- High-definition platforms require explicit cell reconstruction: pipelines such as Bin2Cell and ENACT combine image-based segmentation with expression similarity to yield per-cell matrices with sharper boundaries, integrating smoothly with downstream typing and CCC analysis.
- Neighbor-aware CCC frameworks (COMMOT, SpatialDM, DeepTalk, and niche-DE) move beyond simple ligand–receptor co-expression to infer directional signaling and neighborhood-dependent responses, providing a mechanistic link between local tissue organization and transcriptional regulation in plants.

## 5. From Tissue Atlases to Stimulus Maps—Case Studies

Plant single-cell and spatial transcriptomics have evolved from proof-of-concept to experimentally actionable resources for dissecting environmental responses. High-coverage sc/snRNA-seq references, combined with capture-based spatial maps (Visium/Visium-HD and Stereo-seq) and targeted imaging (smFISH and HCR-FISH), now support robust label transfer, deconvolution of mixed features, and neighborhood-aware inference of cell-to-cell communication. Crucially, multiomics depth (snATAC-seq and multiome) links transcriptional states to putative regulatory elements, formulating testable, detailed hypotheses.

### 5.1. Developmental and Organ Atlases

**Figure 3:**
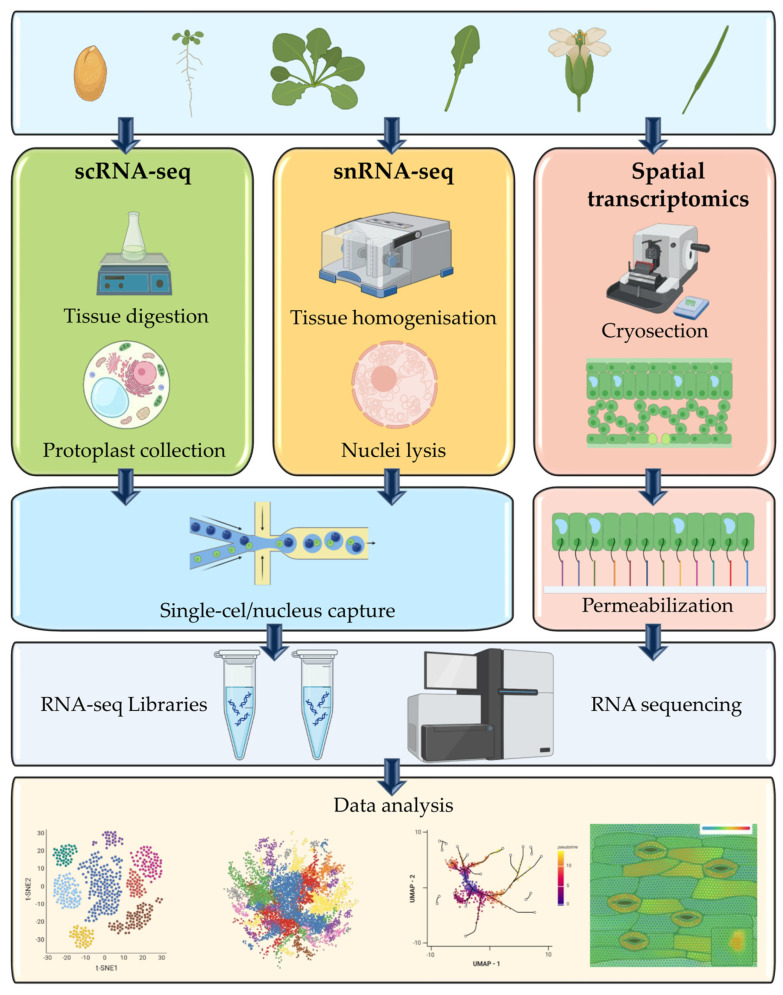
Overview of plant single-cell and spatial transcriptomic workflows. Schematic representation of experimental pipelines used to profile gene expressions at cellular and subcellular resolution in plants. Representative organs (seed, seedling, leaf, rosette, flower, silique) illustrate typical sources of material protoplast-based single-cell RNA sequencing (scRNA-seq), single-nucleus RNA sequencing (snRNA-seq), and spatial transcriptomics. In scRNA-seq, tissues undergo enzymatic digestion to remove cell walls and release protoplasts, whereas in snRNA-seq tissues are homogenized and nuclei are isolated by gentle lysis. Spatial transcriptomics starts from cryo-sectioned tissue slices mounted on capture arrays, followed by controlled permeabilization to release mRNAs in situ. sc/snRNA-seq approaches are typically based on droplet microfluidic capture of individual cells or nuclei, whereas capture-based spatial transcriptomics relies on barcoded arrays that capture transcripts in situ, followed by RNA-seq library preparation and high-throughput sequencing. Downstream analyses include dimensionality reduction and clustering for cell-type identification, trajectory inference for reconstructing developmental or stress-response lineages, and spatial mapping of gene expression patterns within intact tissue architecture. Together, these workflows provide complementary views of plant cell identity, state transitions, and spatial organization. This figure was edited with BioRender (accessed via web application on app.biorender.com).

Integrated developmental/organ atlases illustrate how plant cell identities emerge, diversify, and interact in space and over time (Figure 3, Table 1).

A recent study of the *Arabidopsis* life cycle atlas profiled 432,919 nuclei across ten developmental stages and generated paired spatial transcriptomic datasets for seeds, seedlings, mature leaves, stems, flowers, and siliques [45]. In total 183 clusters (655 subclusters) were annotated, 75% of which were supported by spatial validation, revealing recurrent cell types that reappeared across organs (e.g., the epidermis and vasculature) along with organ-specific heterogeneity and polarity-defined subpopulations [45]. An interactive web viewer (*Arabidopsis* Developmental Atlas) provides stage-resolved embeddings and spatial maps, turning this resource into a community reference for label transfer and hypothesis generation [138].

A multi tissue, multiomics atlas in *Glycine max* extends this paradigm to crops. By integrating snRNA-seq, snATAC-seq and spatial data across ten tissues, the soybean atlas identified 103 distinct cell types and 303,199 accessible chromatin regions (ACRs), ~40% of which showed cell type-specific accessibility enriched for transcription factor motifs [22]. Coordinated activation of the *DOF11* motif containing sucrose transporters in late peripheral endosperm illustrated how cis-regulatory logic underpins developmental trajectories in the embryo and endosperm; conserved regulatory networks linking nodule nitrogen fixation to root cortex programs further demonstrated the power of combining transcriptomic and chromatin layers [22].

In *Zea mays*, a Stereo-seq atlas of the developing maize ear primordium produced a 6 × 6 mm high-resolution spatial map that resolved 12 spatially ordered cell types, including four newly defined populations in the inflorescence meristem [56]. This atlas traces the developmental trajectories underlying reproductive architecture and highlights key transcription factors controlling inflorescence patterning, with data and visualizations made accessible via the Maize Development Ear Spatial Transcriptome Atlas (MDESTA) portal to facilitate deconvolution and neighborhood analysis benchmarks in monocots [139].

These atlases now underpin “stimulus maps” that track how environmental cues reshape local programs. In *Arabidopsis,* life cycle datasets integrate hormone-dependent programs (e.g., morphogenesis of apical hooks) and secondary metabolic pathways allowing spatial comparisons of tissue organization before and after perturbations such as altered light or nutrient status and setting expectations for neighborhood-level dynamics [45]. In soybean analysis of cell type-specific ACRs and motif architectures, cis-elements and transcription factors that serve as sentinels for abiotic or biotic stress are identified, whereas spatial baselines provide controls for niche-resolved differential expression [22]. In maize ears, region-specific gene programs offer a scaffold for studying how temperature or photoperiod perturbations remodel meristem microdomains without disrupting overall organ-level geometry [22,56]. In each case, atlas-grade references, deconvolution, and neighbor-explicit CCC tools, combined with targeted imaging for orthogonal validation, support a systematic transition from static anatomy to spatially constrained, causal physiology.

A complementary example is carbon-concentrating metabolism. Several *Portulaca* species combine canonical C4 photosynthesis with inducible crassulacean acid metabolism (CAM), maintaining carbon gain as water availability decreases [140,141]. Spatially resolved transcript profiling in *P. oleracea* revealed that nocturnal CO_2_ fixation (*PEPC*, malate synthesis and storage) occurs predominantly in mesophyll cells, whereas the daytime decarboxylation machinery resides in bundle sheath (BS) layers, which is consistent with CAM-derived malate feeding into the C4 network during the day [134]. Co-expression modules in which the CAM and C4 genes covary indicate shared control points rather than independent circuits, and genomic analyses suggest that gene duplications have produced carboxylation/decarboxylation paralogs deployable in distinct diurnal phases and tissues [142]. Second, genomic analyses suggest that gene duplications in *P. oleracea* produce copies of carboxylation and decarboxylation components that can be expressed in different diurnal phases and tissues, making it easier to combine cycles that would otherwise conflict with each other [143,144]. For spatial omics, this system illustrates how dissociated snRNA-seq references can be mapped onto Visium HD or Stereo-seq to resolve mesophyll versus BS contributions under drought, with deconvolution quantifying the co-occupancy of CAM-primed mesophyll and BS decarboxylation states and bin-to-cell reconstruction sharpening mesophyll–BS boundaries. Natural variation in inducible CAM among *Portulaca* accessions further permits comparative spatial analyses of C4–CAM integration under water deficit [141].

### 5.2. Plant–Microbe Interactions

Plant–microbe interactions are highly structured across cell layers and neighborhoods. In *Medicago truncatula* roots inoculated with *Rhizophagus irregularis*, Serrano et al. integrated scRNA-seq and spatial RNA-seq with dual-kingdom alignment to construct a co-transcriptomic “map” of arbuscular mycorrhizal (AM) symbiosis [83]. This approach resolved infected and uninfected cortex cells and defined stage-specific states from early penetration through arbuscule maturation and decay. Spatial voxels enriched for fungal transcripts colocalized with the phosphate transporter *MtPT4,* a hallmark of arbusculated cells, and GO analyses of arbuscule clusters highlighted signaling, transmembrane transport and lipid metabolism as key nutrient-exchange processes. Fungal transcripts for secreted effectors and sugar/amino-acid transporters delineate the symbiont’s nutrient acquisition machinery. snRNA-seq, which avoids protoplast artifacts and better recovers fragile arbusculated cells, provides stage-specific marker sets that can be projected onto spatial maps and used to design targeted imaging panels (e.g., HCR-FISH), yielding a dual-linkage atlas of AM symbiosis [83].

Similar strategies can be generalized to nodulation. An integrated nodule–root atlas in soybean combined snRNA-seq with spatial transcriptomics and molecular cartography to resolve interdigitated infected and uninfected cortical populations, revealed transitional infected subtypes at the infection–fixation boundary and functional specialization of initially uninfected cells as nodules matured [120,145]. snRNA-seq resources from earlier stages supplied the developmental context needed to align determinate *Lotus japonicus* nodules, where spatial mapping across successive stages identified *LjNLP3* as a Nin-Like Protein coordinating the differentiation–maturation transition [146]. A common workflow emerges: constructing a high-coverage nuclear reference for nodules and adjacent roots, transferring labels to spatial data and deconvolving capture features, then using spatially enriched markers to design imaging panels that validate boundaries and subcellular localization; finally, comparing species and nodule types to distinguish conserved versus lineage-specific transitions and to test the influence of positional variables (e.g., vascular proximity) on transitional states.

Pathogenesis studies similarly exploit integrated references. In *Arabidopsis* leaves infected with *Pseudomonas syringae*, single-cell and snMultiome analyses, anchored by spatial transcriptomics and multiplex RNA imaging, revealed spatially compartmentalized “immune microneighborhoods” [68,115,147,148]. A rare PRIMER cell population concentrated in immune foci has distinctive regulatory features, including enhanced chromatin accessibility at trihelix factor *GT-3A* binding sites and elevated *GT-3A* expression; PRIMER cells are surrounded by bystander cells expressing long-distance signaling genes such as *ALD1* and *FMO1*, which are central to systemic acquired resistance [68]. Genetic perturbation of *GT-3A* altered susceptibility and salicylic acid pathway activation, thereby validating the regulatory module inferred from spatially constrained trajectories. Label transfer from snRNA-seq to spatial data anchored immune states within the leaf architecture. Neighborhood analyses quantified PRIMER enrichment near substomatal cavities, the canonical pathogen entry sites, and helped explain why adjacent cells adopt distinct roles during infection [68,115].

### 5.3. Abiotic Stresses and Environmental Factors

The spatial transcriptomics of rice roots revealed that drought adaptation comes from anatomically precise programs rather than uniform, organ-wide responses. Spatial atlases of coleoptilar nodes and root tips in upland and irrigated rice genotypes revealed *High-Mobility Group* Box 1 (*HMGB1*) as a central regulator that promotes crown-root elongation and thickening. *HMGB1* acts as a chromatin facilitator that maintains accessibility at nutrient- and stress-responsive loci, priming them for rapid, spatially specific activation [149,150]. Comparative single-cell and spatial profiling of roots grown in natural soil versus gel showed that the outer layers (epidermis, exodermis, and cortex) undergo the strongest reprogramming in soil. This includes the induction of wall remodeling, suberin/lignin deposition, and defense pathways coordinated by abscisic acid (ABA), which is released from phloem cells. This reveals a phloem-to-exodermis communication axis that is weakly engaged in gel-grown roots [151].

High-resolution multiome analyses of *Arabidopsis* root tips exposed to osmotic stress have directly linked changes in chromatin accessibility at individual cis-regulatory elements to subsequent transcriptional responses and lineage trajectories [21]. Shifts in accessibility in lineage-initial cells preceded transcriptional activation and biased downstream fate decisions. For example, these shifts redirected the epidermal bifurcation toward trichoblast identity and altered progression in the quiescent center. Rather than inducing a uniform “stress program,” osmotic stress selectively modulates enhancer logic and fate decisions within specific tissues. This provides a mechanistic view of stress adaptation that is inherently spatial and cell type specific.

### 5.4. Key Takeaways

- Integrated plant atlases that combine sc/snRNA-seq, chromatin accessibility and spatial transcriptomics have moved from proof-of-concept to experimentally actionable references, supporting label transfer, deconvolution and neighborhood-aware analyses.
- Life-cycle and multi-tissue atlases in *Arabidopsis*, soybean and maize provide anatomically grounded maps of cell types and trajectories, together with cis-regulatory landscapes, which can be reused to study diverse developmental and stress contexts.
- These atlases now underpin “stimulus maps” that resolve how environmental cues (light, nutrients, temperature, water) remodel specific microdomains and cell neighborhoods rather than inducing uniform organ-wide responses.
- Spatial omics in *Portulaca* demonstrates how C4 and inducible CAM are temporally and spatially integrated across mesophyll and bundle sheath layers, providing a template for analyzing complex metabolic partitioning under drought.
- Dual-kingdom and nodulation atlases (AM symbiosis, soybean and *Lotus* nodules) reveal how combining snRNA-seq with spatial anchoring reveals rare and transitional states at host–microbe interfaces and identifies candidate regulators that are obscured in bulk data.
- Pathogenesis studies in *Arabidopsis* leaves reveal immune “microneighborhoods” organized around rare PRIMER cells with distinctive regulatory programs, surrounded by bystander cells primed for long-distance signaling, illustrating how spatial context shapes defense roles.
- Spatial transcriptomics under abiotic stress (rice and *Arabidopsis* roots) indicates that adaptation is driven by anatomically precise, enhancer-level modulation of fate decisions and barrier properties, highlighting the need to interpret stress responses in a cell- and tissue-resolved framework.

## 6. Pitfalls and Specifics of the Plant Data

Plant single-cell and spatial transcriptomics present unique challenges that are not typically encountered in animal systems. If default pipelines are applied without adaptation, they can bias the results. For example, rigid cell walls and abundant photosynthetic organelles can influence dissociation efficiency, read composition, and ambient contamination. Moreover, large, polyploid, and repetitive genomes can complicate mapping, quantification, and annotation.

Protoplast-based scRNA-seq introduces two major sources of distortion: rapid transcriptional reprogramming triggered by cellulase–pectinase digestion and handling, and selective recovery of cell types that can be digested and loaded efficiently. Consensus guidelines emphasize that digestion markedly alters the “transcriptional status” and that many tissues and species are recalcitrant, so protoplast libraries can be both stressed and compositionally biased [18]. Single-nucleus RNA sequencing (snRNA-seq) mitigates these issues by bypassing wall digestion and limiting exposure to harsh conditions. However, it yields fewer unique molecular identifiers (UMIs) and genes per barcode, and it captures a nuclear rather than a cytoplasmic snapshot. Therefore, the nuclear and cellular transcriptomes are not interchangeable. Nevertheless, nuclei often provide a more balanced representation and clearer boundaries in chloroplast-rich leaves, mature stems, and other rigid tissues [18]. Refinements, such as exploiting chloroplast autofluorescence during sorting to deplete plastid-rich particles, improve nuclear read alignment and gene detection without reintroducing the stress of protoplasting [152].

In permeabilization-based spatial transcriptomics, the lateral diffusion of mRNA can cause “spot swapping,” whereby transcripts released from one location bind to neighboring features, blurring histological borders. The extent of diffusion depends on the platform’s chemistry, the tissue type, and the permeabilization conditions [82]. Probabilistic methods, such as the SpotClean model, treat observed counts as mixtures of native and swapped transcripts. These methods have been shown to increase marker specificity and stabilize clustering in complex tissues. In practice, careful optimization of section integrity, fixation, and permeabilization is combined with an empirical estimate of a “bleeding index,” and spatial decontamination is applied before boundary-sensitive analyses, such as domain calling, spatial differential testing, or ligand–receptor inference [153,154,155].

Organellar RNA requires plant-specific quality control. Community guidelines caution against applying mammalian-style mitochondrial cut-offs. Instead, they advocate for tissue- and species-specific thresholds that reflect normal plastid and mitochondrial activity [18]. Poly(A)-based capture enriches for nuclear mRNA but also retrieves plastid transcripts because plant organelles polyadenylate RNAs during decay. Thus, chloroplast-derived reads in poly(A) libraries often represent genuine biology rather than artifacts [156,157]. Elevated plastid fractions in photosynthetic tissues may indicate active physiology or decay intermediates. They should be interpreted via cell-type markers, spatial context, and physiology, rather than being removed by default.

snRNA-seq can reduce plastid carryover by gating nuclei on chloroplast autofluorescence before DAPI staining. This improves nuclear alignment and transcript quantification in leaf tissues while reducing UMI complexity. Single-cell and spatial atlases of *Arabidopsis* leaves demonstrate robust chloroplast transcription in photosynthetic populations, highlighting that plastid signals are often physiological [55,158,159]. Repeating key inferences across alternative organelle-read filters and cell- and nucleus-derived inputs in sensitivity analyses is recommended.

Polyploidy and incomplete annotations introduce additional complexity. In polyploid genomes, the high sequence similarity between homeologous and paralogous loci causes multimapping. This complicates UMI deduplication and inflates spurious differences when only uniquely mapped reads are retained. EM-based pipelines, such as ALEVIN-Fry, which retain equivalence-class information and explicitly model UMI collisions, reduce these artifacts while remaining scalable to large plant datasets [160]. Homeolog-aware approaches are crucial for preserving dosage-biased expression and sub-genome asymmetry. Curated references and single nucleotide polymorphism (SNP) panels can resolve homeolog expression in 3′ RNA-seq, as demonstrated in wheat [161,162]. Cell-type identities can also be transferred from well-annotated species. Markers are anchored in orthology, and placements are validated against untargeted spatial maps [5]. Spatial assays introduce additional constraints because probe-based chemistries may hybridize to multiple homeologs, unless SNPs and copy numbers are explicitly considered. Empirical validation and cultivar-matched single-cell or nucleus references improve specificity. Deconvolution results should include uncertainty estimates reflecting both ambient RNA and homeolog ambiguity [136,163,164].

Ambient RNA in droplets is a pervasive confounding factor in plant sc/snRNA-seq. Extracellular transcripts from lysed or damaged cells mix into cellular profiles, decreasing marker specificity and increasing apparent similarity between populations. Deep generative models, such as CellBender, infer ambient distributions from empty and cell-containing droplets. Bayesian mixture models, such as DecontX, estimate per-cell contamination fractions via cluster structure and gene-level priors. Empirical approaches exploit expression asymmetries or cross-species “barnyard” designs to calibrate ambient loads and benchmark decontamination [31,165,166]. Droplet-based snRNA-seq is also affected by cytoplasmic carryover and debris. Tailoring filtering and decontamination to nuclear data improves annotation fidelity in solid tissues. Since background removal measurably impacts differential expression, integration, and downstream modeling, recent evaluations recommend conducting explicit sensitivity analyses and transparently reporting ambient profiles and per-cell contamination estimates alongside final plant atlases [167].

### Key Takeaways

- Plant single-cell and spatial data cannot use generic animal pipelines: rigid walls, abundant plastids and polyploids, repetitive genomes create plant-specific dissociation, mapping and QC artifacts that must be handled explicitly.
- Protoplast scRNA-seq induces stress reprogramming and selectively recovers easily digested cell types, whereas snRNA-seq sacrifices UMI depth for more balanced cell-type representation and fewer dissociation artifacts, especially in rigid, chloroplast-rich tissues.
- In spatial assays, mRNA diffusion and “spot swapping” blur borders; carefully tuned fixation/permeabilisation plus model-based decontamination (e.g., SpotClean) should precede domain calling, spatial differential expression (DE) and ligand–receptor analyses.
- Organellar reads, particularly from chloroplasts, often reflect true biology; tissue- and species-specific thresholds, together with markers and spatial context, are preferred over rigid mitochondrial-style cut-offs, and key results should be tested across alternative organelle filters.
- Polyploidy and incomplete annotations require homeolog-aware quantification: EM-based mappers (e.g., alevin-fry), curated SNPs and orthology-anchored markers help preserve sub-genome asymmetry, whereas spatial probe design and deconvolution must account for multimapping and reporting uncertainty.
- Ambient RNA contamination is ubiquitous in plant sc/snRNA-seq and affects nuclei as well as cells; deep generative, Bayesian and empirical decontamination approaches should be adapted to nuclear data, with ambient profiles and per-cell contamination estimates reported alongside atlas outputs.

## 7. Horizons: From Maps to Trait Modification

Single-cell and spatial transcriptomics (SRTs) transforms cellular atlases into actionable blueprints for crop improvement. They do this by linking regulatory peaks, genes, and cell-type transcriptional regulatory networks (TRNs) to their precise anatomical context under control and stress conditions. This is particularly true when RNA + ATAC “multiome” profiling is combined with root atlases that integrate sc/snRNA-seq and spatial validation [5,21]. Cell-type-specific cis-regulatory element (CRE) maps and single-cell gene regulatory networks (GRNs) now allow editing targets to be prioritized directly from these atlases. In maize and rice, single-cell ATAC-seq and regulome resources have identified cell-type-specific open chromatin regions that are enriched with Genome-wide association study (GWAS) variants and linked to candidate genes. This highlights enhancer-rich regions as natural substrates for precision editing and marker design [168,169]. Multiome profiling further strengthens this logic by establishing links between peaks and genes in nuclei undergoing developmental or stress-induced transitions and by reconstructing tissue-type transcriptional regulatory networks (TRNs). In practice, atlas-informed pipelines rank candidate cis-regulatory elements (CREs) by their activity in the causal tissue or state, by the editability of their motif architecture for base or prime editing and CRISPRa/i, and by their overlap with genetic signals (GWAS/expression quantitative trait locus (eQTL)). Spatial readouts then confirm that the perturbations operate within the intended domain [21,168,169,170]. Precise cis-regulatory editing provides a more subtle way to modulate gene expression than coding knockouts do, thereby reducing pleiotropy. In tomato and cereal crops, promoter edits at loci such as *KLUH/CYP78A* or *OsSTOMAGEN* generate a graded allelic series that shifts fruit size or stomatal density in a stepwise manner rather than producing binary phenotypes [171,172]. Moreover, deletion of a defined cis-motif in the *OsNAS2* promoter increased Zn and Fe accumulation in rice grains without compromising yield, indicating nutrient-focused dosage tuning [173]. Cell type-targeted CRISPRa/i achieves place specificity by driving localized pathway activation. It can also be coupled with chemically inducible systems to provide temporal control during critical developmental windows [174,175]. In polyploid crops, homeolog-aware design enables allelic series that mirror sub-genome-biased expression patterns revealed by atlases, as demonstrated in allotetraploids [176]. Transitioning from spatially informed hypotheses to edited lines necessitates delivery methods that are rapid and genotype-flexible. Four complementary strategies—developmental regulator (DR)-assisted transformation, de novo meristem induction, haploid inducer-mediated genome editing (HI-Edit/IMGE), and viral/mobile guide prototyping supported by precision editors—can shorten this engineering loop. DRs, such as WUSCHEL (WUS), BABY BOOM (BBM), and PLETHORA (PLT), increase regeneration competence and expand the transformable genotype space. Transient DR expression in intact tissues (de novo meristem induction) enables editing without prolonged tissue culture. This reduces somaclonal variation and line-generation time, providing a practical bridge from atlas-derived targets to edited genotypes [177,178]. HI-Edit couples haploid induction with genome editing. It introduces edits during fertilization and recovers haploids that carry the edits. These haploids are then doubled to create fixed diploids. Because the edits are delivered via an inducer line, the efficiency is largely independent of the recipient’s transformability. Maize case studies have demonstrated that HI-Edit can shorten timelines and simplify allele stacking for breeding applications [179,180]. Plant RNA and DNA viral vectors enable the rapid prototyping of guide designs directly in plants. pLX-based systems and related platforms deliver single-guide RNAs (sgRNAs) or full CRISPR components into Cas-expressing hosts. These systems can reach reproductive tissues and generate heritable edits with minimal tissue culture. Engineered restricted-motion vectors can broaden the host range and limit systemic spread. These same vectors can deliver CRISPR activation (CRISPRa) or inhibition (CRISPRi) guides to test whether candidate enhancers or transcription-factor nodes identified from single-cell or multiome maps activate the expected cell-type program. This allows researchers to confirm the effectiveness of the guides prior to committing to stable edits [181,182,183]. Base editors and prime editors are particularly well suited for the small sequence changes required for CRISPR engineering. Recent advances in plants, including prime editing guide RNA (pegRNA) stabilizers, engineered reverse transcriptases, and optimized expression strategies, have improved prime editing efficiency. Moreover, PAM-related Cas variants and refined editor scaffolds now support motif-level base editing across diverse genomes in a homeolog-aware manner [184,185,186,187,188]. During the selection and validation stage, single-cell and spatial transcriptomics directly link gene activity to tissue architecture. This provides “place-aware” markers for breeding and functional engineering [2]. Unlike classical QTL mapping, atlases pinpoint the tissues, cell types, and boundaries in which candidate genes and CREs are active [189]. They also sharpen the prioritization of causal variants, especially noncoding polymorphisms with effects confined to critical cellular niches and supply spatial markers for in situ validation of genome edits [147,190]. These resources provide a mechanistic link between sequence variation and phenotype by filtering QTL candidates according to their expression in relevant cell types and stages, as well as by connecting GWAS variants to their likely target genes using cell type-specific chromatin and eQTL maps [169,191,192]. Spatial markers at pathogen interfaces provide tissue-resolved readouts for disease resistance editing and enable the monitoring of defense modules in precise epidermal or apoplastic domains [129,147]. For nutritional traits, promoter edits, such as the *OsNAS2* example, link DNA variation to micronutrient loading and yield-related traits in grain tissues. These proximal molecular phenotypes can accelerate early selection and complement field-level evaluation [173].

### Key Takeaways

- Single-cell and spatial transcriptomics (including the RNA + ATAC multiome) turn descriptive atlases into cell type-resolved maps of cis-regulatory logic, enabling direct prioritization of editing targets from the CRE and GRN/TRN frameworks.
- Enhancer- and promoter-centered cis editing (CRISPRa/i and small cis changes) enables graded, tissue-specific and cell type-specific tuning of expression, reducing pleiotropy and supporting dosage- and place-aware trait modification, including in polyploids.
- Atlas-based pipelines rank CREs by activity in the causal tissue/state, editability (base/prime editing) and overlap with GWAS/eQTL signals, whereas spatial readouts verify that perturbations act in the intended anatomical domain.
- A toolkit of delivery routes—DR-assisted transformation, de novo meristem induction, HI-Edit/IMGE and viral/mobile-guide systems—shortens the path from the spatial hypothesis to the edited line and opens recalcitrant genotypes to editing.
- Base and prime editors, PAM-related Cas variants and optimized pegRNA/editor designs now enable motif-level, homeolog-aware cis editing across complex genomes, matching the resolution of single-cell/multiome regulatory maps.
- Single-cell and spatial atlases provide “place-aware” markers that refine QTL-to-gene assignment, prioritize noncoding variants and offer tissue- and interface-specific readouts (e.g., infection sites, grain loading zones) for validating edits and accelerating selection.

## 8. Conclusions

A plant’s functional organization is determined by its complex tissue layers, in which spatial positioning determines the direction and impact of molecular signals and cellular responses. Integrating single-cell and spatial transcriptomics into this architecture has fundamentally changed our ability to explain mechanisms and study complex traits. Three overarching principles can be derived from recent advances.

First, the spatial context should be regarded as the central axis of interpretation, since identical changes in transcription levels yield divergent biological outcomes depending on their anatomical location, whether in the epidermis, endoderm, bundle sheath, or companion cells of the phloem.

Second, the resolution of cellular heterogeneity afforded by dissociative single-cell approaches comes at the expense of coordinates, whereas spatial transcriptome mapping retains positional fidelity but sometimes conflates cell identities owing to pixel size or spot mixing. Their integration—through label transfer and spot deconvolution—enables a more accurate reconstruction of tissue mosaics and, in the multiomics era, facilitates the direct linking of chromatin accessibility and gene expression dynamics within the same nucleus, illuminating the regulatory events delimiting domains or revealing non-cell-autonomous signals. Methodologically, plant-specific workflows are needed: enzymatic protoplasting can distort the cell-type composition and induce rapid stress responses, whereas single-nucleus protocols mitigate these artifacts but typically at the cost of reduced transcript capture. Spatial assays require vigilant correction for transcript diffusion and ambient RNA to support quantitative analyses at tissue boundaries, whereas organelle-derived reads and cell wall-induced mapping errors require refined thresholds and reference strategies—particularly in polyploid species. These are not peripheral challenges but primary constraints that must be addressed from experimental design onward.

Despite the rapid pace of innovation, current spatial technologies still impose significant limitations. Most capture-based platforms offer near-rather than actual single-cell resolution in complex organs. High-definition assays at the cellular scale are costly, data-intensive, and not yet commonplace for large-scale comparative studies. However, throughput and gene-detection sensitivity are still limited. Transcript dropout, low per-spot UMI counts, and restricted panel sizes constrain the detection of rare states and subtle regulatory shifts in imaging-based methods. These constraints underscore the need for robust cross-species label transfer and comparative atlas construction, particularly in polyploid crops, where homeologous redundancy and incomplete annotations obscure orthology and cell-type correspondence. Future efforts will require harmonized reference frameworks, orthology-aware markers, and homeolog-resolved spatial maps. These maps will enable comparisons of cell identities, regulatory modules, and stress programs across genotypes, species, and ploidy levels. This will transform isolated atlases into a coherent, evolutionary, and agronomically relevant atlas network.

Third, the availability of single-cell and spatial atlases enables actionable biological inference. High-resolution maps of cell-type regulatory landscapes and cis-regulatory elements support the prioritization of targets for precise genome editing. In contrast to coding knockouts, cis-modifications offer advantages such as the capacity for spatial and temporal specificity and the mitigation of pleiotropy. Innovations such as cell type-restricted CRISPRa/i, novel delivery approaches, and advanced base or prime editing platforms have streamlined the iterative process from hypothesis to trait engineering. Validating the anatomical specificity and physiological effects of such edits is now feasible via the use of spatial markers and in situ molecular readouts. The most transformative studies will pursue atlasing and functional testing under real agricultural conditions, integrating diverse omics to model cell- and tissue-level programs, and deploying spatially resolved validation. Harmonized community standards for data reporting—including sample preparation, RNA and organelle handling, and computational deconvolution—increase reproducibility and meta-analysis. Ultimately, public access to integrated portals featuring single-cell, spatial, and multiomics data will facilitate the cross-comparison and application of findings across species and organs. This apparent spatial paradigm extends beyond the realm of plant diversity cataloging, thereby enabling rational, tissue-targeted engineering of crop phenotypes with lasting value for plant breeding and sustainable agriculture.

## Figures and Tables

**Table 1:** List of single-cell/nuclear and spatial transcriptomics studies in different plant species.

Species	Tissue/Cell Type	Technology	Reference
*Arabidopsis thaliana*	Root	scRNA-seq Spatial (10× Visium)	[20,23,107,108,109] [110]
	Shoot apex	scRNA-seq	[111]
	Leaf	scRNA-seq Spatial (MERFISH)	[55,112,113,114,115] [114]
	Stomata	scRNA-seq	[116]
	Multiple tissues	snRNA-seq Spatial (MERFISH)	[6,45] [45]
	Epidermal cell	Spatial (scStereo-seq)	[55]
*Brassica pekinensis*	Leaf	scRNA-seq	[117]
*Catharanthus roseus*	Leaf	scRNA-seq	[118,119]
*Glycine max*	Root	scRNA-seq Stereo-seq	[120] [120]
*Gossypium bickii*	Cotyledon	scRNA-seq	[121]
*Hevea brasiliensis*	Leaf	scRNA-seq	[122]
*Hordeum vulgare*	Seed	Spatial (10× Visium)	[81]
*Lotus japonicus*	Root	scRNA-seq	[123]
*Medicago truncatula*	Nodule	scRNA-seq Spatial (10× Visium) Spatial (10× Xenium)	[124] [83] [125]
*Nicotiana attenuata*	Corolla cell	scRNA-seq	[124]
*Oryza sativa*	Root	scRNA-seq	[126,127,128]
	Leaf	scRNA-seq	[128,129]
*Pisum sativum*	Shoot	scRNA-seq	[130]
*Populus trichocarpa*	Xylem	scRNA-seq	[131,132]
*Populus* spp.	Stem	Spatial (10× Visium)	[133]
*Portulaca oleracea*	Leaf	Spatial (LCM, 10× Visium)	[134]
*Solanum lycopersicum*	Callus (shoot)	scRNA-seq Spatial (Stereo-seq) Spatial (10× Visium)	[135]
*Triticum aestivum*	Grain (early development)	Spatial (BMKManu)	[136]
*Zea mays*	Ear	scRNA-seq	[56]
		Spatial (Stereo-seq)	[56]
	Shoot apical meristem	scRNA-seq	[137]

## Data Availability

No new data were created or analyzed in this study. Data sharing is not applicable to this article.

## Supplementary Materials

The following supporting information can be downloaded at https://www.mdpi.com/article/10.3390/ijms262411819/s1.
