# Supplementary material for: Why “Where” Matters as Much as “How Much”: Single-Cell and Spatial Transcriptomics in Plants
Source: Int J Mol Sci. 2025 Dec 7;26(24):11819. doi: 10.3390/ijms262411819 (PMC12732828; doi:10.3390/ijms262411819)
Supplement: Supplementary file 1 [file ijms-26-11819-s001.zip › Table S1.pdf]

Table S1. Comparative Landscape of Single-Cell and Single-Nucleus RNA-seq Platforms

Platform/apparatus	Capture principle	Typical throughput	Input (plants)	UMI	Sensitivity (genes/cell)	Doublet rate (typical)	Recommended reads/cell	Equipment required
10x Genomics Chromium (Controller/X/iX), 3'/5'	Microfluidic droplets (GEMs)	500–20,000 per channel; higher with HT/multiplex	Protoplasts or nuclei (snRNA-seq)	Yes	~1,500–5,000 (species/tissue dependent)	~1% per 1,000; ~7–8% at 10,000	25k–50k (nuclei often ~25k)	Chromium instrument, chips, NGS
BD Rhapsody	Microwell cartridge with barcoded beads	100–40,000 per cartridge	Cells or nuclei	Yes	Similar to 10x for 3' counting	~2–3% at 10k; ~8–10% at 40k load	20k–50k	Rhapsody scanner/cartridges, NGS
Drop-seq (custom or Nadia/Dolomite)	Droplet microfluidics with barcoded beads	Tens of thousands per run	Protoplasts; nuclei via DroNc variants	Yes	Moderate; cost-effective	Poisson-dependent; similar to 10x at matched loading	25k–50k	Droplet generator, pumps, beads, NGS
inDrop (1CellBio)	Droplet microfluidics, hydrogel barcoded beads	Tens of thousands	Protoplasts (limited plant reports)	Yes	Moderate	Poisson-dependent	25k–50k	inDrop system, NGS
Seq-Well / Seq-Well S3	Picowell array sealed by membrane	~2,000–10,000 per array (scalable with arrays)	Cells; nuclei variants possible	Yes	Moderate	Low with proper loading	25k–50k	Arrays, plates, membrane, NGS
Fluidigm C1 (legacy)	Microfluidic capture sites (size-gated)	96–800 per chip	Cells (size-dependent)	No (SMARTer chemistry)	High, full-length	Low	0.3–1.0M	C1 instrument/chips, NGS
Smart-seq2/3 (plate-based)	FACS into plates; full-length RT-PCR	96–384 per plate	Cells or nuclei (Smart-seq3-n)	Smart-seq2: No; Smart-seq3: Yes	Very high, full-length isoforms	Very low	0.2–1.0M	FACS, liquid handling, NGS
CEL-Seq2 (plate-based)	3' IVT amplification with UMIs	96–384 per plate	Cells (sorted)	Yes	Moderate–high for 3' counting	Very low	50k–200k	FACS, liquid handling, NGS

SPLiT-seq (combinatorial)	Split-pool barcoding (no capture device)	100k–1M	Fixed cells or nuclei	Yes	Low–moderate per cell	Low (no droplets)	25k–50k (often less)	Standard lab plates/reagents, NGS
sci-RNA-seq / scifi-RNA-seq (combinatorial)	Combinatorial indexing	100k–1M+	Nuclei or cells (fixed)	Yes	Low–moderate per cell	Low	25k–50k (often less)	Standard lab plates/reagents, NGS
