# Supplementary material for: Why “Where” Matters as Much as “How Much”: Single-Cell and Spatial Transcriptomics in Plants
Source: Int J Mol Sci. 2025 Dec 7;26(24):11819. doi: 10.3390/ijms262411819 (PMC12732828; doi:10.3390/ijms262411819)
Supplement: Supplementary file 1 [file ijms-26-11819-s001.zip › Table S2.pdf]

Table S2 Comparative landscape of spatial transcriptomics platforms

Platform/Method	Principle & Main Features	Spatial Resolution	Transcript Coverage	Typical Plant Applications	Key Technical Notes
10x Genomics Visium	Oligo-barcoded slide arrays, NGS	~55 μm (classic); 2–10 μm (HD)	Whole transcriptome (polyA RNAs)	Arabidopsis, maize, soybean, barley, wheat, tomato	Requires custom sample prep, cryo-sectioning, plant permeabilization
Stereo-seq (BGI/MGI)	DNA nanoball-patterned chips, NGS	<1 μm, high definition (bins 0.5–1 μm)	Whole transcriptome (NGS)	Maize, rice, caryopses, endosperm, tubers	Needs silicone gaskets, large FOV, specialized plant sample prep
Slide-seq v2	DNA-barcoded beads on coverslip (random array), NGS	~10 μm	Whole transcriptome	Arabidopsis, ovules, various tissues, cost-efficient	Flexible chemistry, demands decoding of bead array spatial layout
High-Definition Spatial Transcriptomics (HDST)	Bead-based array, lith. defined wells	2 μm	Whole transcriptome	Leaves with fine architectures (e.g., Arabidopsis)	Enhanced spot definition with BayesSpace for resolution boost
Laser Capture Microdis-section (LCM)+RNA-seq	Laser-excised microregions into tubes for library prep	~10–50 μm (tissue dependent)	Region-specific, can target small areas, can be deep	Various seeds, roots, leaves	Labor-intensive, low throughput (high-resolution for target locus)
GaST-seq (Grid-assisted) Spatial Transcriptomics	Micro-scale grid sampling, manual section	~300 μm	Regional transcriptomics	Arabidopsis leaf, biotic stresses	Simple, cost-effective for spatial profiling, lower spatial resolution
smFISH, HCR-FISH	Imaging-based, probe hybridization (amplified/non-amplified FISH)	Single-molecule, sub-cellular (1–2 μm)	Targeted (gene panels), Strong for rare transcripts	Root/quiescent centers, whole-mount samples (Arabidopsis, maize)	Multiplexed, requires optical clearing, gene set design
MERFISH	Imaging, combinatorial probe rounds	Single-molecule, sub-cellular (1 μm)	High multiplex (100–1000+) Targeted panels	Arabidopsis developmental atlas Plant immune cell mapping	Error-corrected barcoding, plant-specific probe optimization
